# Minimally Invasive Surgery to Remove a Broken and Retained Epidural Catheter Fragment

**DOI:** 10.7759/cureus.25255

**Published:** 2022-05-23

**Authors:** Sarthak Walia, Tushar Pisal, Anirudh Kandari, Purvam Jivrajani

**Affiliations:** 1 Department of Orthopaedics and Traumatology, Dr. D. Y. Patil Medical College, Hospital and Research Centre, Pune, IND

**Keywords:** spine surgery, total hip replacement, minimally invasive surgery, fragment, epidural catheter

## Abstract

Epidural anaesthesia (EA) has consistently been used for treatments affecting the pelvis, lower limbs, lower abdomen, and perineum; however, it is progressively employed as a single anaesthetic or supplement to general and spinal anaesthesia for a broader range of procedures. The retention of a broken epidural catheter piece is an uncommon but well-known complication. In this report, we present a 30-year-old male with avascular necrosis (AVN) of the hip who was referred for total hip replacement (THR). An epidural catheter had been placed at the presumed L2-3 interspace to administer EA. The catheter had been set too deep and it broke on extraction with the Tuohy needle, leaving a fragment inside. The patient was then given general anaesthesia and the planned procedure of THR was done in the lateral position. The patient was then shifted to the prone position to remove the retained fragment of the epidural catheter by a minimally invasive spinal surgery (MISS). Right-sided L2 laminotomy was done, as the epidural catheter was inserted from the right side, to retrieve the broken fragment without any added postoperative neurological complications. MISS may be attempted by experienced surgeons for the removal of a retained fragment of the epidural catheter from the spinal canal before adhesion as a safe option.

## Introduction

Avascular necrosis (AVN) of the hip is a distressing, crippling disorder that occurs when intraosseous bone marrow pressure rises due to pathological conditions like steroid intake, haemostatic disorders, or trauma causing an increase in pressure transmitted to minute blood vessels of the bone, resulting in poor blood flow to the bone [1]. AVN history is uncertain. Merle D'Aubigné et al. followed up on 90 instances with AVN [2]. A collapse occurred in 20% of them after one year, and a meagre 25% averted collapse after three years of onset [2].

Age, gender, laterality, use of pre-reduction traction, lack of proximal femoral ossific nucleus, adductor tenotomy, preliminary/history of hip abduction bracing, and hip abduction angle in the cast have all been identified as probable risk factors for AVN in previous investigations [3-11]. Brougham et al. studied 184 participants undergoing closed reduction of 210 hip dislocations and determined AVN in 47% of cases [6]. It was later proved that the risk of AVN was unaffected by gender, laterality, age, adductor tenotomy, or previous use of hip abduction orthosis.

Although several treatment approaches, such as stem cell transplant and core decompression, have been explored for early AVN, total hip replacement (THR) had consistently been proven to be the most efficient in treating advanced AVN of the femoral head [12,13]. However, the early functional outcome of THR in AVN is still debatable, especially as published evidence revealed a higher requirement for early hip revision procedures [14].

For the administration of epidural anaesthesia (EA), a catheter tube is inserted through the skin into the spine's epidural space, which is made of polyethylene. It is used for anaesthesia/analgesia in a range of clinical operations and acute conditions. The catheter provides access to the epidural space for the injection of pain-relieving medications, such as local anaesthetics or opioids. However, difficulties associated with the same are well-known to be relatively frequent. There are rare cases of epidural catheters becoming entangled or breaking in the medical literature. In the current report, a case of a broken epidural catheter is presented while extraction during the procedure.

## Case presentation

**Figure 1:**
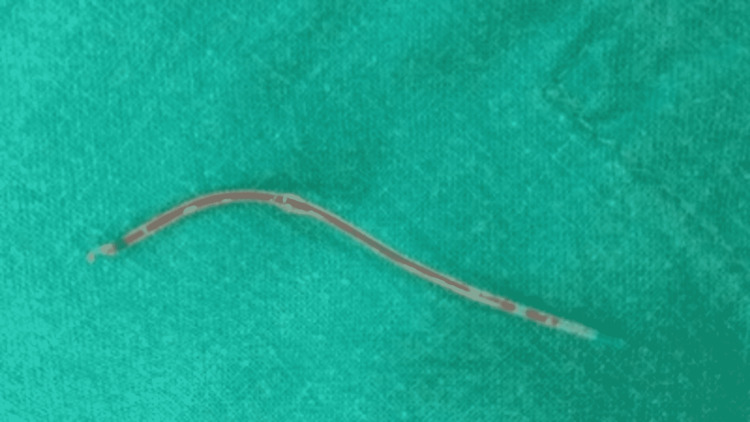
Epidural catheter retrieved after surgical exploration.

A 30-year-old male with AVN of the hip was referred for THR. An epidural catheter had been placed at the presumed L2-3 interspace to administer EA. It had been placed too far, thus on extraction with a Tuohy needle, it broke, leaving a fragment inside. The patient was then given general anaesthesia and the planned procedure of THR was done in the lateral position. After the scheduled surgery, the patient was shifted to the prone position to remove the retained fragment of the epidural catheter by a minimally invasive spinal surgery (MISS). The level of insertion of the epidural catheter was marked according to the needle prick site under the guidance of C-arm imaging. The epidural catheter was inserted cranially from the site of insertion from the right side of the spinous process. The incision was taken accordingly and serial dilatation was done using dilators. A tube of 19 mm diameter was inserted over the dilator and the position was confirmed under fluoroscopy. The epidural catheter was not visible under fluoroscopy imaging. Right L2 laminotomy was done, the ligamentum flavum was removed, and the dura was exposed. The epidural catheter was localized in the right paraspinal gutter and pulled out by using a blunt nerve hook. The broken epidural catheter fragment was removed by MISS with minimum soft tissue injury without any added postoperative neurological complications (Figure 1).

The patient was mobilized from day one post-operatively with mild back pain. Back pain persisted for six weeks after surgery, which was managed with back exercises. At one year follow-up, there was no back or hip pain.

## Discussion

When inserting or withdrawing a catheter, EA is not impervious to complications. Epidural catheterization is a relatively secure technique with a low risk of complications. Breaking of an epidural catheter in the epidural space during insertion or removal is an uncommon problem with not many documented occurrences, especially during the placement of an epidural catheter [15].

Shearing of the catheter may be due to the catheter breaking into two fragments when withdrawing through the Tuohy needle, catching unsharpened needle barbs because of applying force or by heavy contact between a bony surface and the tip of the needle, when a portion of the catheter protruded from the tip, or weak catheters owing to manufacturing defects, or looping, knotting, or entanglement by resistance from anatomical parts like vessels, nerve roots, fascia, vertebral processes, facet joint, and posterior vertebral arches, etc., or excessive threading leading to entanglement or twisting between the epidural space and skin [15-18]. In the abovementioned case, the catheter broke in two parts while withdrawing through the Tuohy needle and the anaesthetist realized it immediately.

Despite the retained catheter being radiopaque, radiological imaging techniques are typically inconclusive in detecting it. This could be owing to the tiny thickness of the epidural catheter as well as the high radiodensity of the surrounding tissue [15]; hence, catheters should be formed of materials that enhance their visibility [19].

If epidural catheter breaks during removal, the fragment must be documented and communicated to patients and families. They must also be reassured regarding the likelihood of neurological issues being low as well as that there are treatment options available for them [16-19].

Even though the neurological sequelae of retained catheters are scarce, several reported cases consider surgical removal in the first place [19,20]. The other possible options in such cases can be either to leave it in place in case of an adult patient and educate the patient about any warning signs, or check with a spine surgeon in case of any neurologic deficit or difficulties [15,20].

By about three weeks, within the epidural space, fibrous tissue forms a barrier around the retained portion of the catheter. However, symptoms are generally seen if the catheter impinges or stretches a nerve, a superimposed infection develops, or, in rare cases, when foraminal stenosis in a patient is presented with lower back pain. Surgical intervention in such cases is a necessity [15]. The catheter can be removed by MISS with fewer complications, minimum soft tissue damage, and better long-term outcome. MISS is advantageous over open surgery and is well mentioned in the literature [20].

Most recorded instances of epidural insertion occur between the L2 and L5 interspaces, perhaps because lumbar epidurals are more common. Catheter material may also contribute, as polyurethane catheters or nylon can be far more durable compared to polyethene or Teflon catheters, while catheters of 19 gauge tend to rupture around the tip at a fixed location.

## Conclusions

To avoid breakage and retention of the epidural catheter, typical procedures for insertion and removal should be effectively implemented regularly. In cases of retained catheters, MISS may be attempted by experienced surgeons for the removal before the adhesions advance.
